# The Structural Versatility of the BTB Domains of KCTD Proteins and Their Recognition of the GABA_B_ Receptor

**DOI:** 10.3390/biom9080323

**Published:** 2019-07-31

**Authors:** Nicole Balasco, Giovanni Smaldone, Luigi Vitagliano

**Affiliations:** 1 Institute of Biostructures and Bioimaging, CNR, Via Mezzocannone 16, 80134 Napoli, Italy; 2 IRCCS SDN, Via Gianturco 113, 80143 Napoli, Italy

**Keywords:** oligomerization, molecular dynamics simulations, protein–protein interactions

## Abstract

Several recent investigations have demonstrated that members of the KCTD (Potassium Channel Tetramerization Domain) protein family are involved in fundamental processes. However, the paucity of structural data available on these proteins has frequently prevented the definition of their biochemical role(s). Fortunately, this scenario is rapidly changing as, in very recent years, several crystallographic structures have been reported. Although these investigations have provided very important insights into the function of KCTDs, they have also raised some puzzling issues. One is related to the observation that the BTB (broad-complex, tramtrack, and bric-à-brac) domain of these proteins presents a remarkable structural versatility, being able to adopt a variety of oligomeric states. To gain insights into this intriguing aspect, we performed extensive molecular dynamics simulations on several BTB domains of KCTD proteins in different oligomeric states (monomers, dimers, tetramers, and open/close pentamers). These studies indicate that KCTD-BTB domains are stable in the simulation timescales, even in their monomeric forms. Moreover, simulations also show that the dynamic behavior of open pentameric states is strictly related to their functional roles and that different KCTDs may form stable hetero-oligomers. Molecular dynamics (MD) simulations also provided a dynamic view of the complex formed by KCTD16 and the GABA_B2_ receptor, whose structure has been recently reported. Finally, simulations carried out on the isolated fragment of the GABA_B2_ receptor that binds KCTD16 indicate that it is able to assume the local conformation required for the binding to KCTD.

## 1. Introduction

Studies carried out in the last decade have demonstrated that members of the KCTD family, proteins containing a Potassium (K) Channel Tetramerization Domain, are involved in fundamental physiopathological processes [1,2,3,4,5]. Although initial investigations have highlighted the involvement of these proteins in brain diseases, more recent studies have indicated that they play roles in many other biological processes. In particular, KCTD11 is involved in the etiology of medulloblastoma [6], KCTD7 in epilepsy [7], KCTD2/KCTD5/INC in sleep homeostasis [8,9,10], KCTD8/12/16 in GABA_B_ receptor (GABA_B_R) modulation [11], while KCTD13 is believed to play an important role in autism [12,13]. Nevertheless, these proteins also play key roles in other processes that include obesity [14], breast carcinoma [15], and SEN syndrome [16,17].

**Figure 1:**
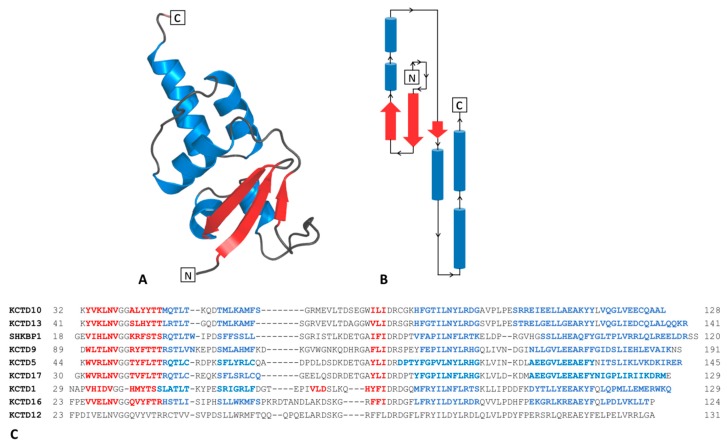
Cartoon representation (**A**) and topology (**B**) of the five α-helices and the three/four-stranded β-sheet motif of the BTB domain. Sequence alignment of the BTB domains of the KCTD proteins considered in this study (**C**). Residues belonging to α-helices and β-strands are colored in blue and red, respectively. For KCTD12, whose structure has not been experimentally determined, the assignment of the secondary structure is not reported on the sequence.

Although the involvement of KCTD proteins in these key processes is well documented, their biochemical function is frequently unknown. From the molecular point of view, KCTDs are multi-domain proteins characterized by the presence of a BTB (broad-complex, tramtrack, and bric-à-brac) domain in the N-terminal region of their sequences [2]. In KCTD proteins the BTB domain presents the canonical structural organization characterized by a single three/four-stranded β-sheet surrounded by five α-helices (Figure 1A,B) [18]. The rest of the sequence of KCTD proteins may be totally unrelated among different members of the family [2]. In general terms, these proteins may be sub-divided in two major groups. The first one includes the members of the family that are able to bind Cullin3 and that likely function as adaptors in Cullin-RING ubiquitin ligases for substrate ubiquitination and degradation [2,3,19,20,21]. In other members, the incapacity to bind Cullin3 is coupled with the ability to bind AP-2α (KCTD1/KCTD15) [22] or GABA_B_R (KCTD8/KCTD12/KCTD16).

Structural data available on these proteins have been rather poor for many years. Indeed, the data were limited to the KCTD5 member whose pentameric structure was determined a decade ago [23]. Very recently, other structural information derived through the application of different approaches became available [19,21,24,25,26,27,28]. These studies have provided some interesting clues on the structure and partnerships of these proteins. Nevertheless, they also opened some puzzling questions. Of particular interest is the observation that BTB domains of these proteins may appear in a variety of oligomeric states (Figure S1). In contrast to the tetrameric states (C4 symmetry) observed in the closest homologs [21,24,27], the BTB domains of KCTD proteins are able to adopt monomeric, tetrameric (222 symmetry), and open/close pentameric states. The functional relevance of these states is debated as some of them could be artefactual. In this scenario, we present here extensive molecular dynamics (MD) simulations on the various oligomeric states of KCTD-BTB domains (Figure S1). Present findings provide interesting insights into the basis of the structural versatility shown by these domains. Moreover, simulations carried out on the BTB domain of KCTD16 (KCTD16^BTB^) and on the C-terminal region of the GABA_B2_ receptor (GABA_B2_R) have provided clues on the molecular mechanism that regulates the recognition between these two biological partners.

## 2. Results

The extensive analyses of the dynamic/structural properties of the BTB domains of the KCTD proteins here reported were undertaken by considering different oligomeric states of these domains (Figure S1). MD simulations were performed on systems with a progressive structural complexity, starting from monomers to end up with pentamers (Figure 1C). The convergence of the trajectories was assessed by computing the root mean square inner product (RMSIP) (Table 1) and the autocorrelation function of the energy (see Materials and Methods for details).

### 2.1. Analysis of the Structure and Stability of the KCTD-BTB Domains in Their Monomeric Forms

#### 2.1.1. The BTB Domain of SHKBP1

The recently reported crystal structure of the BTB domain of SHKBP1 (SHKBP1^BTB^) surprisingly showed that this domain is monomeric in the crystal state [21] despite its established role in protein oligomerization. Although this finding could be biased by the removal of the C-terminal region of the protein, we here evaluated the stability of the SHKBP1^BTB^ monomer in a crystal-free environment. 

**Figure 2:**
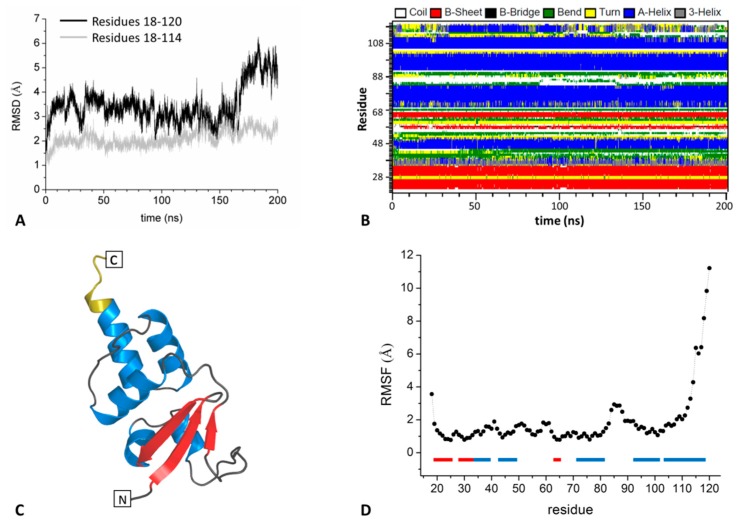
Structure stability of SHKBP1^BTB^ throughout the simulation: C^α^-based RMSD values of trajectory structures calculated against the starting crystallographic model (**A**), time evolution of the secondary structure content (**B**). The RMSD values have been calculated on the whole structure (residues 18-120, black) or by excluding the last six residues at the C-terminus (residues 18-114, grey). Cartoon representation of SHKBP1^BTB^ (**C**), α-helices and β-strands are colored in blue and red, respectively. The last six residues at the C-terminus are in yellow. C^α^-based RMSF values of residues of SHKBP1^BTB^ calculated in the 50–200 ns trajectory region (**D**)**.** The protein secondary structure elements are reported as bars (α-helices in blue and β-strands in red).

A collective analysis of the indicators commonly used to evaluate the stability of the system along the trajectory indicates that SHKBP1^BTB^ is stable as monomer in the simulation timescale (Figure 2A,B). Indeed, secondary structure elements are rather well preserved (Figure 2B). During the simulation, the formation of a transient extra β-strand within the sheet is observed in the α2-β3 region. Notably, a similar four-stranded β-sheet is present in the crystallographic structure of the BTB domain of KCTD1 (KCTD1^BTB^) (PDB ID: 5BXB) [27]. This indicates that the size of the β-sheet of these proteins is somehow variable. The inspection of the RMSD values indicates that the system undergoes significant structural rearrangements at the very early stage of the simulation (Figure 2A). As commonly observed in MD studies, this rapid increase of the RMSD values of the trajectory structures compared to the crystallographic model can be attributed to the relaxation of the system that is transferred from the crystal state to a solution-like environment. However, the RMSDs reach rather constant values (~3–4 Å) within the first ~20 ns of the simulation. It is worth mentioning that the sudden increase of the overall RMSD values observed at ~160 ns is due to the local reorganization of the last six residues at the C-terminus. This is not surprising since this region of the protein does not make contacts with the rest of the protein body (Figure 2C). In line with this observation, a shortening of the fifth helix emerges from the analysis of the secondary structure (Figure 2B). We also evaluated the dynamics of this domain by monitoring the RMSF values calculated on the C^α^ atoms in the 50–200 ns trajectory region. As shown in Figure 2D, most of the protein regions display rather limited motions (RMSF values of 1–1.5 Å) despite the monomeric state. Flexible regions correspond to loops and to the very end C-terminal residues (RMSF values > 6 Å) that do not make contact with the main body of the protein. 

#### 2.1.2. The BTB Domain of KCTD1, KCTD5, KCTD9, KCTD10, KCTD13, KCTD16, and KCTD17

**Figure 3:**
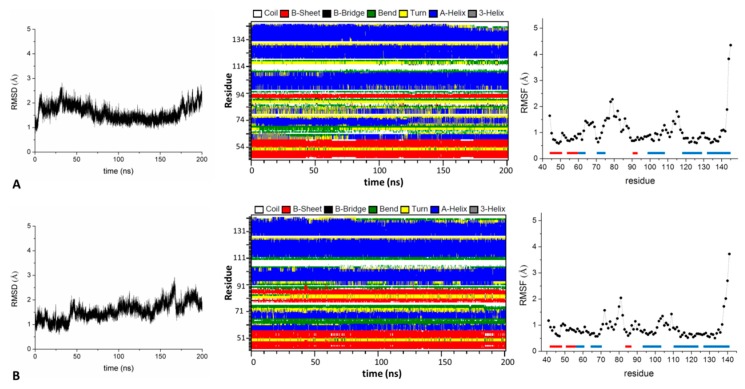
Structure stability of KCTD5^BTB^ (**A**) and KCTD13^BTB^ (**B**) monomers throughout the MD simulations: C^α^-based RMSD values of trajectory structures computed against the starting model, time evolution of the secondary structure content, C^α^-based RMSF values computed in the equilibrated region of the trajectory (50–200 ns). The protein secondary structure elements are reported as bars (α-helices in blue and β-strands in red).

The results obtained for SHKBP1^BTB^ monomer prompted us to perform similar analyses on the other KCTD-BTB domains whose structures were reported in higher oligomeric states. In particular, we extracted monomers from tetramers (KCTD10 and KCTD13) and close (KCTD1, KCTD5, KCTD9, and KCTD17) or open (KCTD16) pentameric states. An overview of the structures obtained in the MD trajectories indicates that all of these monomers are rather stable in the simulation timescale (200 ns) (Figure 3 and Figure S2). In Figure 3, we show as representative examples the RMSD values calculated against the starting crystallographic models, the secondary structure and the RMSF values obtained for the BTB domains of KCTD5 (KCTD5^BTB^) and KCTD13 (KCTD13^BTB^) monomers that were derived from pentameric or tetrameric assemblies, respectively. Similar results have been obtained for the other monomers as reported in Figure S2 of the Supplementary Material. In all cases, an equilibrated state is reached within ~50 ns. Moreover, highly flexible regions are confined to loops and to the end of the polypeptide chain as observed for SHKBP1^BTB^. Collectively, these findings indicate that the BTB domains may share a common property related to the stability of their monomeric species, so far crystallographically observed only for SHKBP1^BTB^.

### 2.2. Analysis of the Structure and Stability of the KCTD-BTB Domains in Tetrameric and Dimeric States

In the next step of our characterization of the dynamic/structural properties of these domains we focused our attention on the tetramers that are formed by the BTB domains of KCTD10 (KCTD10^BTB^) and KCTD13 (KCTD13^BTB^) [21]. Moreover, since these assemblies are made by the association of dimers (the tetramer is a dimer of dimers) that present interfaces also observed in pentameric forms, we also checked the stability of this basic building block. 

**Figure 4:**
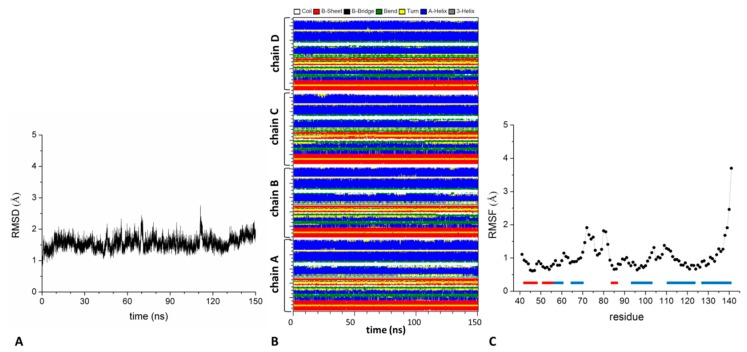
Structure stability of KCTD13^BTB^ tetramer throughout the MD simulation: C^α^-based RMSD values of trajectory structures against the starting model (**A**), time evolution of the secondary structure content (**B**), C^α^-based RMSF values computed in the 50–150 ns trajectory region (**C**). The protein secondary structure elements are reported as bars (α-helices in blue and β-strands in red).

The inspection of the RMSD values computed against the starting crystallographic model (~1.5 Å for t > 10 ns) highlighted a remarkable rigidity of this assembly that is also confirmed by the preservation of all secondary structure elements (Figure 4). In line with this observation, very low RMSF values are observed (<1.5 Å) with the exception of the α2-β3 loop region and the C-terminal end (RMSF values in the interval 2–4 Å). The same behavior is also exhibited by the closely related KCTD10^BTB^ tetramer (Figure S3). Finally, the MD simulation performed on the dimeric building block that constitutes the asymmetric tetramer confirmed the high stability and rigidity of this basic architecture (Figure S4).

Although the biological role of the tetrameric assembly is undermined by the observation that both KCTD10^BTB^ and KCTD13^BTB^ switch toward the formation of pentameric state in the presence of their biological partner (Cullin 3) [21], these findings highlight the versatility of these domains to oligomerize in different architectures. 

### 2.3. Analysis of the Structure and Stability of the KCTD-BTB Domains in Pentameric States

#### 2.3.1. Close States (KCTD1^BTB^, KCTD17^BTB^, and KCTD9^BTB^)

MD simulations carried out on the close pentameric states exhibited by KCTD1^BTB^, KCTD9^BTB^, and KCTD17^BTB^ indicate that these assemblies are very stable in the simulation timescale in line with the results we previously reported for the pentameric state of KCTD5^BTB^ [19,25,29] (Figures S5–S7). A comparative analysis of the RMSF values of the same chains in the monomeric and pentameric states did not highlight significant differences. This finding corroborates the observation that BTB domains of KCTD proteins are also rather rigid in the monomeric state. 

#### 2.3.2. Open States (KCTD1^BTB^ and KCTD16^BTB^)

The crystallographic characterization of some BTB domains (KCTD1^BTB^ and KCTD16^BTB^) has led to the discovery of peculiar forms in which open pentamers are observed [21,27]. We also confirmed the occurrence of these unexpected states by negative stain electron microscopy [24]. Although the biological significance of these states is yet to be assessed, we here evaluated their dynamic properties. For both KCTD1^BTB^ and KCTD16^BTB^, no major structural reorganization was observed (Figure S8). Secondary structure elements are rather well-preserved in the simulation timescale for both proteins. On the other hand, KCTD16^BTB^ is endowed with higher average RMSF values (~1.5 Å) than those (<1 Å) observed for KCTD1^BTB^ (Figure S9A). A deeper inspection of Figure S9C indicates that this is due to the higher fluctuations (RMSF values in the range 1.5–4 Å) exhibited by residues belonging to the external chains that delimitate the gap compared with those of the internal domains (RMSF values < 1 Å). It is worth noting that this distinct behavior of the different (internal/external) chains is essentially limited to the loop regions in the simulation of the open KCTD1^BTB^ (Figure S9B). 

**Figure 5:**
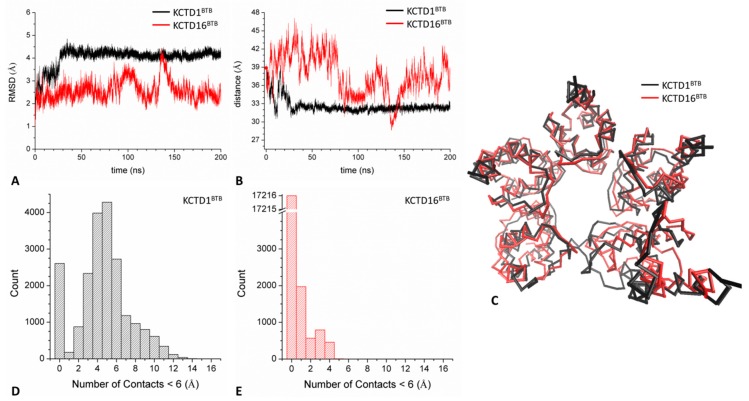
C^α^-based RMSD values of trajectory structures against the starting model obtained in the MD simulations performed on KCTD1^BTB^ and KCTD16^BTB^ open pentamers (**A**). Distance between the centers of mass of the two external domains that delimitate the gap in KCTD1^BTB^ and KCTD16^BTB^ open pentamers (**B**). The red dot indicates the common starting value (~39 Å). Superimposition of the C^α^-trace of KCTD1^BTB^ and KCTD16^BTB^ average structures computed in the equilibrated region of trajectories (50–200 ns) (**C**). Distribution of the number of atoms of the two external domains that are within 6.0 Å in KCTD1^BTB^ (**D**) and KCTD16^BTB^ (**E**).

A further inspection of the gap present within the open pentamers highlights other significant differences. A comparative analysis of the RMSD values computed against the starting models shows that both KCTD1^BTB^ and KCTD16^BTB^ systems undergo an initial structural transition in the first 40–50 ns of simulations (Figure 5A). Interestingly, larger RMSD values are observed for KCTD1^BTB^ compared to KCTD16^BTB^. On the other hand, KCTD16^BTB^ reaches a sort of equilibrated region characterized by much larger fluctuations of the RMSDs although the average value (~2.5 Å) is essentially preserved. This is not surprising as some variations of the open pentamer of this BTB domain have also been highlighted through comparison of the crystallographic structures detected starting from different crystal forms ([26,28], in particular Figure S1 of [28]).

Altogether, present results indicate that KCTD1^BTB^ undergoes a larger structural transition compared to KCTD16^BTB^ that leads to the formation of more rigid states. To get deeper insights into the reorganizations of these systems, we performed additional analyses of the evolution of the gap that characterizes the open states. As shown in Figure 5B, in KCTD1^BTB^, the two domains that delimitate the gap get quite close to generate a sort of closed state. Indeed, the distance between the centers of mass of these two domains stabilizes at ~32 Å from 39 Å of the starting model. Although this state is not coincident with the crystallographic one, the external domains make several contacts that stabilize it. In contrast to KCTD1^BTB^, in KCTD16^BTB^ a dynamic open state is preserved throughout the simulation as shown by the superimposition of the average protein structures computed in the equilibrated region (50–200 ns) of trajectories (Figure 5C). Furthermore, the number of atoms of the two domains that are within 6.0 Å is significantly higher (up to 13) in KCTD1^BTB^ compared to KCTD16^BTB^ (up to 4) (Figure 5D,E). Altogether, these findings indicate that KCTD1^BTB^ and KCTD16^BTB^ have a different propensity to preserve the open state in the MD simulation. To better characterize this different propensity, we evaluated analogies and differences in the interactions that stabilize the domain–domain interfaces in the different systems. We focused our attention into the interface formed by the central domain i.e., the farthest chain from the gap. As shown in Figure S10, these interfaces contain a significant larger number of hydrogen bonds in KCTD16^BTB^ compared to KCTD1^BTB^ (on average 17 *versus* 11). It is likely that the strongest interactions present in KCTD16^BTB^ prevent its evolution toward a close state, thus, preserving its functional open state (see below). 

#### 2.3.3. Mixed Open Pentamer Formed by KCTD12^BTB^ and KCTD16^BTB^

**Figure 6:**
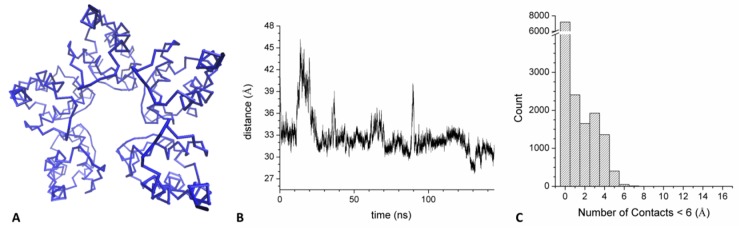
Average structure of KCTD12-16^BTB^ hetero-pentamer computed in the 50–150 ns region of the MD trajectory (**A**). Distance between the centers of mass of the two external domains that delimitate the gap (**B**). Distribution of the number of atoms of the two external domains that are within 6.0 Å (**C**).

One intriguing feature of KCTD proteins is that some specific members of the family are able to mix in the same oligomeric assembly. Among others, of particular interest is the functional role exhibited by the hetero-oligomer formed by KCTD12 and KCTD16 [30]. Since this ability of KCTDs has never been explored at atomic level, we here generated a mixed pentameric assembly formed by the BTB domains of KCTD12 and KCTD16 (KCTD12-16^BTB^) (see Methods for details). This model was used as a starting structure for an MD simulation run. The analysis of the resulting trajectory indicates that, despite the sequence heterogeneity of its constitutive polypeptide chains, this assembly is rather stable in the simulation timescale as it preserves the secondary structure content of all chains (Figure S11A). Interestingly, the mixed oligomer retains its open pentameric structure thus reproducing the behavior shown by the KCTD16^BTB^ pentamer (Figure 6A). Indeed, although the terminal domains of this assembly come closer than those of KCTD16^BTB^ (Figure 6B), they do exhibit a significant mobility as indicated by the fluctuations of the RMSD values (Figure S11B). Moreover, the RMSF values of residues of the external domains (chains A and E) are significantly higher (>1.5 Å) than those exhibited by those belonging to the central chains (<1 Å) as previously observed for KCTD16^BTB^ (Figures S9C and S11C). Also, the number of atoms of the two external domains that come within 6.0 Å is rather limited and is clearly lower (6 *versus* 13) than those found in KCTD1^BTB^ that, as previously shown, evolves toward a close state in the simulation (Figure 6C and Figure 5D).

### 2.4. Mechanism of GABA_B2_R Recognition by KCTD16

While this work was in progress, two independent structures of the complex formed by the BTB domain of KCTD16 with C-terminal peptide(s) of GABA_B2_ receptor were reported [26,28]. Although the complexes described in these papers share important structural analogies, the interactions between the protein and the peptides are not completely coincident (Figure S12). This is likely due to the different length of the GABA_B2_R peptide used in the two studies (residues 881-913 or 895-909). In order to get further insights into the recognition process of these proteins, we performed MD simulations on this system. In particular, to achieve a dynamic view of the interaction between KCTD16^BTB^ and the GABA_B2_R peptide (residues 881-913), we performed an MD simulation on the complex. Moreover, since we have experimentally shown that the C-terminal region of the peptide adopts a limited but significant level of secondary structure [31], we also carried out an MD run on the isolated peptide to get information on its intrinsic structural properties. 

**Figure 7:**
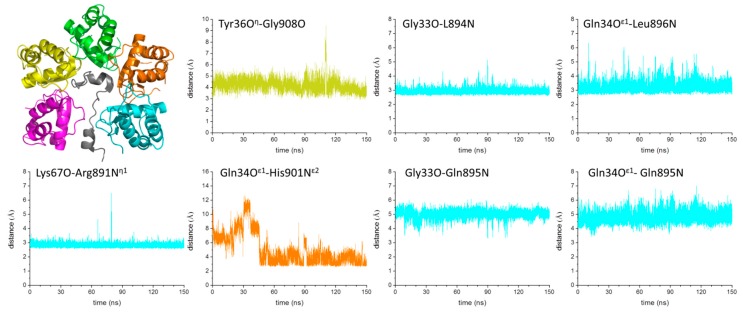
Cartoon representation of the crystal structure of the complex between KCTD16^BTB^ and GABA_B2_R peptide (PDB ID: 6M8R) used as starting model in the MD simulation. Time evolution of the distances between pairs of atoms involved in the formation of H-bonding interactions in the complex. The H-bonds between atoms Tyr36O^η^-Gly908O, Gly33O-L894N, Gln34O^ε1^-Leu896N, and Lys67O-Arg891N^η1^ that are present in the starting crystallographic model are conserved throughout the simulation. The H-bonds between atoms Gln34^Oε1^-His901^Nε2^, Gly33^O^-Gln895^N^, and Gln34^Oε1^-Gln895^N^ that are not present in the starting structure but are present in the structure of the complex between KCTD16^BTB^ and the GABA_B2_R peptide encompassing residues 895-909 are formed in the simulation. Plots are colored to identify the chains of KCTD16^BTB^ that interact with the peptide (in grey).

The simulation performed on the complex unravels the tight binding of the two partners as no detachment is observed in the trajectory structures. As indicated by the time evolution of the RMSD values computed against the starting model, the system reaches a stable state (RMSDs ~2.5 Å) after ~40 ns (Figure S13A). Secondary structure elements of both KCTD16^BTB^ and GABA_B2_R peptide are essentially retained in the simulation timescale (Figure S13B,C). Interestingly, in contrast to what we observe for the isolated KCTD16^BTB^ pentamer (Figure S9C), no significant differences can be observed in the RMSF values of residues belonging to the external chains compared to those of the internal ones (Figure S13D). Evidently the formation of the complex makes the terminal domains of KCTD16^BTB^ open pentamer more rigid. A global analysis of the protein–peptide interactions suggests a dynamic behavior of the KCTD16^BTB^-GABA_B2_R recognition process. Several H-bonding interactions detected in the starting crystallographic model are conserved in trajectory structures (Tyr36O^η^-Gly908O, Gly33O-L894N, Gln34O^ε1^-Leu896N, Lys67O-Arg891N^η1^) (Figure 7) whereas others are either sporadic or lost (Val35^N^-Val910^O^, Glu28O^ε2^-Ser913O, Glu102O^ε2^-Tyr903O^η^, Ser69N-Arg891O, Ser69O^γ^-Gln889O^ε1^, Asp91O^δ1^-Glu886O^ε12^, and Asp91O^δ2^-Arg890N^η1^) (Figure S14). Interestingly, we also found interactions that were not present in the starting crystallographic structure but that were present in the structure of the complex between KCTD16^BTB^ and the GABA_B2_R peptide encompassing residues 895-909 (Gln34O^ε1^-His901N^ε2^, Gly33O-Gln895N, and Gln34O^ε1^-Gln895N) (Figure 7).

**Figure 8:**
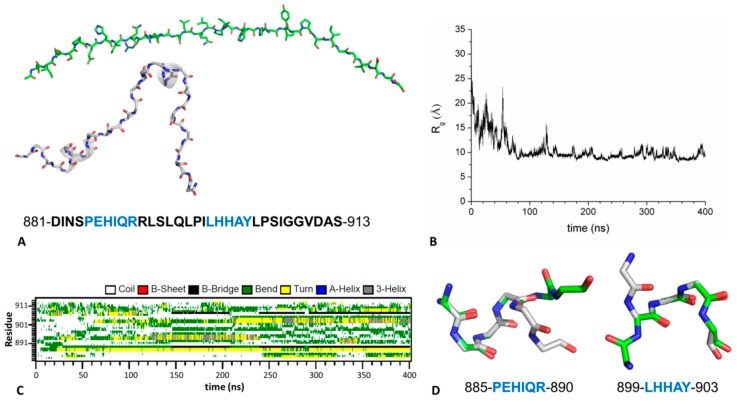
Representation of the fully extended structure (φ = −120°, ψ = 130°, ω = 180°) (green) of the GABA_B2_R peptide (residues 881-913) used as starting model in the MD simulation of the isolated peptide. The aminoacid sequence and the structure (grey) of the peptide extracted from the crystallographic complex with KCTD16^BTB^ (PDB ID: 6M8R) are also shown. Residues 885-890 and 899-903 which adopt a helical conformation are colored in blue in the sequence (**A**). Time evolution of the radius of gyration R_g_ (**B**) and of the secondary structure content (**C**) of GABA_B2_R peptide. Representative examples of the conformational states detected in the simulation (green) superimposed to the crystallographic model (grey) in the regions 885-890 and 899-903 (**D**).

The simulation carried out on the isolated peptide was started using a fully extended conformation (see Methods for details) (Figure 8A). As shown by the analysis of the gyration radius (R_g_), a collapse (from ~29 to ~10 Å) of the model occurs after ~80 ns of the simulation (Figure 8B); this is coupled with strong deviations of the trajectory structures from the starting fully extended conformation (Figure S15A). Interestingly, some small secondary structure elements (3_10_ helices) start to appear after 100 ns (Figure 8C). Interestingly, these correspond to the helical regions of the peptide observed in the complex (residues 885-890 and 899-903, Figure 8A). As shown in Figure 8D, the local conformation of the peptide in the regions 885-890 and 899-903 observed in some trajectory structures is nearly coincident with that detected in the crystallographic structure of the complex. It is also worth mentioning that, in line with our previous experimental data [31], the fraction of structures in which these helical states are observed is rather limited (~14%) (see also Figure 8C). Nevertheless, as shown in Figure S15B,C almost all of trajectory structures of these regions present very low RMSD values compared to the crystallographic structure (<1.0 Å). These observations indicate that the peptide is intrinsically able to adopt the conformation required to bind KCTD16^BTB^_._

## 3. Discussion

BTB domains, also denoted as POZ domains, are widespread structural modules that are present in a variety of multidomain proteins [18,32,33]. They are typically considered as domains specifically devoted to homo-oligomerization and more rarely involved in hetero-oligomerization. Their ability to self-associate to allow homo-oligomerization of multidomain proteins is observed in voltage-gated potassium channels. These proteins possess a cytoplasmic BTB domain (T1) that favors the functionally important tetramerization of the membrane channels [34]. Although BTB domains of KCTD proteins share significant sequence and structural similarities with the T1 domains [2], their role in homo-oligomerizaton appears to be less stringent. Indeed, recent independent studies have highlighted unsuspected oligomeric associations of these domains [21,24,27]. These peculiar findings may be, at least in part, due to the fact that these characterizations have been conducted on isolated KCTD-BTB domains. Nevertheless, we believe that is of interest that domains that are considered to be the driving factors for homo-oligomerization easily dissociate generating a variety of different states (monomers, tetramers, and open/close pentamers). In this framework, we here report extensive MD analyses of KCTD BTB domains in different oligomeric architectures.

The characterization of a variety of KCTD-BTB domains in their monomeric state indicates that they are quite stable in the simulation timescale. Interestingly, individual residues of these monomers present fluctuations similar to those detected in higher oligomeric states. In general, the larger flexibility of the main body of this domain was detected for the α2-β3 linker. Since this loop is, in some KCTDs, implicated in Cullin3 binding [19], the observed mobility may have a functional role. 

The stability of the individual monomers of KCTD BTB domains is in line with their versatility in adopting different architectures. In this scenario, it is not surprising that larger and likely non-functional states such as dimers and tetramers with a 222 symmetry are also stable. These findings also suggest that other oligomeric assemblies, not yet reported, formed by these proteins are in principle possible. In line with our previous characterization of the pentameric state of KCTD5 protein [19,25], the compact BTB pentamers of KCTD1, KCTD9, and KCTD17 are also stable in the simulation timescale. These findings well agree with the idea that close pentameric assemblies are the functional states of the sub-class of KCTD proteins that bind Cullin3 [20,21]. 

The characterization of KCTD1 and KCTD16 open pentameric states highlights that these assemblies are endowed with different dynamic/structural properties. Indeed, in the simulation timescale, the KCTD1^BTB^ open pentamer evolves toward a rather closed and rigid state whereas KCTD16^BTB^ presents a highly dynamic behavior and essentially retains its open structure. Interestingly, while this work was in progress, two independent investigations have shown that the open pentamer is the functional form of KCTD16^BTB^ as it anchors the C-terminal region of the GABA_B2_ receptor, a key partner of KCTD16, using the gap between the external domains present in this assembly [26,28]. By showing that this gap is preserved in KCTD16^BTB^ trajectory structures, present MD results indicate that the highly dynamic BTB domain of this protein retains the open binding-prone functional state that was experimentally observed in the complex with the GABA_B2_R peptide. 

A very interesting result reported in recent literature is the observation that mixed KCTD12/KCTD16 assemblies are functional [30]. We here analyzed the structural and the dynamic behavior of a mixed KCTD12^BTB^/KCTD16^BTB^ pentamer. The results of the MD simulation indicate that it behaves as the KCTD16^BTB^ homopentamer with an open structure that allows the binding of the receptor. The ability of KCTD12/KCTD16 to form mixed species is not unique in this protein family [14,30,35]. KCTD proteins may use this property as a mechanism to modulate their activity. The observed structural versatility of the BTB domains, which seem to be stable also in their monomeric forms, may be important to achieve this goal as the formation of mixed species will likely proceed through intermediates with non-canonical oligomeric structures. 

The determination of the structure of the complex between KCTD16^BTB^ and the GABA_B2_ receptor gave us the possibility to extend our MD analyses to these systems. The dynamic view of the assembly shows that this complex is stabilized by interactions that were alternatively present in one of the two crystallographic structures [26,28]. More interestingly, we evaluated the intrinsic structural properties of the C-terminal region of the GABA_B2_ receptor to estimate whether the isolated peptide was able to adopt the conformations experimentally observed in the complex.

Notably, the present analysis of the intrinsic conformational properties of the isolated peptide clearly indicates that two of its regions present a limited, but significant, propensity to adopt the helical structure that is the motif it assumes in the complex with KCTD16^BTB^ [26,28]. This indicates that the ensemble of structural states of the GABA_B2_R peptide contains conformations that are intrinsically structured to bind KCTD16^BTB^. Notably, present MD data indicate that, as found in other peptide-protein interactions [36,37], both partners of the KCTD16^BTB^-GABA_B2_R complex are endowed with an intrinsic propensity to adopt conformations experimentally detected in this hetero-assembly. 

Collectively, our findings here highlight the role that structural versatility of BTB domains plays in the architecture and functionality of this common module present in all members of the emerging class of KCTD proteins. 

## 4. Material and Methods

### 4.1. Notations and System

As revealed by the crystallographic structures of KCTD proteins so far reported, the BTB domain of these proteins may adopt a remarkable variety of oligomerization states, including monomer (SHKBP1), two-fold rotationally symmetric tetramers (KCTD10, and KCTD13), close pentamers (KCTD5, KCTD9 and KCTD17), and open pentamers (KCTD1 and KCTD16) (Figure S1). For these proteins, MD simulations were carried out considering both the monomeric state of the domain and the oligomeric architecture experimentally observed. The crystal structure of the monomeric BTB of SHKBP1 (SHKBP1^BTB^, PDB ID: 4CRH) has been considered [21]. For the other KCTDs, the structure of a single BTB chain was used as starting model in the simulations performed on protein monomers. In detail, monomeric models have been generated from the following PDB codes: 5BXB for KCTD1 [27], 3DRZ for KCTD5 [23], 5BXH for KCTD9 [27], 5FTA for KCTD10 [21], 4UIJ for KCTD13 [21], 5A15 for KCTD16 [21], and 5A6R for KCTD17 [21]. Additional MD simulations were performed on KCTD oligomers using the experimental crystallographic structures as starting models: KCTD10^BTB^ tetramer (PDB ID: 5FTA), KCTD13^BTB^ tetramer (PDB ID: 4UIJ), KCTD1^BTB^ close pentamer (PDB ID: 5BXB), KCTD17^BTB^ close pentamer (PDB ID: 5A6R), KCTD9^BTB^ close pentamer (PDB ID: 5BXH), KCTD1^BTB^ open pentamer (PDB ID: 5BXD), and KCTD16^BTB^ open pentamer (PDB ID: 5A15). A simulation run was also carried out considering the dimeric building block of KCTD13^BTB^ tetramer (PDB ID: 4UIJ) as model. Moreover, a mixed open pentamer (KCTD12-16^BTB^) composed of three chains of KCTD16^BTB^ (chains A, C, and E) and two of KCTD12^BTB^ (chains B and D) built using the open KCTD16^BTB^ pentamer (PDB ID: 5A15) as template was used as starting model in a novel simulation. For open pentamers, the two BTB chains (A and E) that delimitate the gap have been denoted as external chains whereas the others (chains B, C, and D) have been denoted as internal chains. 

Two crystallographic structures of the BTB domain of KCTD16 in complex with the peptide corresponding to the C-terminal domain of the human GABA_B2_ receptor have been recently reported (Figure S12) [26,28]. The structure containing the GABA_B2_R peptide encompassing residues 881-913 has been used as starting model in the MD simulation (PDB ID: 6M8R). A fully extended structure (φ = −120°, ψ = 130°, ω = 180°) of the GABA_B2_R peptide encompassing the same portion was modeled and used as starting structure in a novel MD simulation. 

### 4.2. MD Protocol

Molecular Dynamics (MD) simulations were performed on KCTD proteins using the GROMACS software package with Amber99sb as force field [38]. The models were immersed in triclinic boxes filled with water molecules (TIP3P water model) and counterions (Na^+^ or Cl^−^) to balance charges. Timescales and several parameters of the simulations (timescale, box dimensions, number of water molecules) are reported in Table 1. The simulations were carried out applying periodic boundary conditions. Systems were first energy minimized for 50,000 steps using steepest descent. Equilibration of each system was first conducted for 500 ps at 300 K temperature (NVT ensemble) and then for 500 ps at 1 atm pressure (NPT ensemble). The Parrinello–Rahman and the Velocity Rescaling methods were used for pressure and temperature control, respectively. The Particle Mesh Ewald (PME) with a grid spacing of 1.6 Å was used to compute the electrostatic interactions [39]. For Lennard–Jones interactions, a cut-off of 10 Å was applied. Bond lengths were constrained using the LINCS algorithm [40]. An integration time step of 2 fs was used. Analysis of MD trajectories was performed by using GROMACS routines and the VMD program [41]. The root mean square inner product (RMSIP) parameter [42], computed between the two halves of the reduced trajectories (starting from 50 ns to the end) considering the protein motions of the C^α^ atoms along the first 10 eigenvectors, was used to check the achievement of an adequate convergence by MD simulations [43,44]. As indicated by the RMSIP values reported in Table 1, all simulations reach a satisfactory level of convergence. A rapid drop of the autocorrelation function of the potential energy as function of time is also observed for all simulations (Figure S16).

## Figures and Tables

**Table 1:** Parameters and statistics of MD simulations.

System	Timescale (ns)	Box Dimensions (nm^3^)	No. of Water Molecules	RMSIP ^a^
SHKBP1^BTB^ monomer	200	5.24 × 5.20 × 7.02	5770	0.78
KCTD1^BTB^ monomer	200	6.43 × 5.22 × 4.96	4924	0.80
KCTD5^BTB^ monomer	200	4.83 × 5.56 × 6.39	5158	0.72
KCTD9^BTB^ monomer	200	5.15 × 4.91 × 6.61	4985	0.68
KCTD10^BTB^ monomer	200	5.23 × 5.00 × 6.69	5284	0.82
KCTD13^BTB^ monomer	200	6.89 × 5.25 × 5.16	5589	0.78
KCTD16^BTB^ monomer	200	6.43 × 6.05 × 5.44	6318	0.72
KCTD17^BTB^ monomer	200	5.95 × 5.23 × 6.53	6245	0.77
KCTD13^BTB^ tetramer	150	7.73 × 7.46 × 9.07	15618	0.65
KCTD10^BTB^ tetramer	150	7.67 × 9.06 × 7.23	14570	0.79
KCTD13^BTB^ dimer	150	7.32 × 6.84 × 6.51	9735	0.78
KCTD1^BTB^ close pentamer	150	8.58 × 6.87 × 8.38	13845	0.60
KCTD9^BTB^ close pentamer	150	8.93 × 9.16 × 6.87	15988	0.49
KCTD17^BTB^ close pentamer	150	8.73 × 9.09 × 8.13	18724	0.60
KCTD1^BTB^ open pentamer	200	7.65 × 9.93 × 8.47	18280	0.67
KCTD16^BTB^ open pentamer	200	7.87 × 10.41 × 8.99	21809	0.72
KCTD12-16^BTB^ open pentamer	150	7.83 × 9.79 × 8.99	19886	0.73
KCTD16^BTB^-GABA_B2_R complex	150	7.84 × 9.62 × 9.07	19624	0.66
GABA_B2_R peptide	400	11.15 × 6.10 × 4.77	10509	0.73

^a^ The RMSIP values have been calculated by dividing the reduced trajectories (starting from 50 ns to the end) in two equivalent halves.

## Supplementary Materials

The following are available online at https://www.mdpi.com/2218-273X/9/8/323/s1. Figure S1. Cartoon representation of the BTB domains of KCTD proteins used as starting models in the MD simulations of monomers, tetramers, dimer, close and open pentamers. PDB codes are reported in bracket. Figure S2. Structure stability of KCTD1BTB (A), KCTD9BTB (B), KCTD10BTB (C), KCTD16BTB (D), and KCTD17BTB (E) monomers throughout the simulations: Cα-based RMSD values of trajectory structures computed against the starting model, time evolution of the secondary structure content, Cα- based RMSF values computed in the equilibrated region of the trajectories (50–200 ns). The protein secondary structure elements are reported as bars (α-helices in blue and β-strands in red). Figure S3. Structure stability of KCTD10BTB tetramer throughout the MD simulation: Cα-based RMSD values of trajectory structures computed against the starting model (A), time evolution of the secondary structure content (B), Cα-based RMSF values computed in the 50–150 ns region of the trajectory (C). The protein secondary structure elements are reported as bars (α-helices in blue and β-strands in red). Figure S4. Structure stability throughout the MD simulation of the dimeric building block that constitutes the asymmetric KCTD13BTB tetramer: Cα-based RMSD values of trajectory structures computed against the starting model (A), time evolution of the secondary structure content (B), Cα-based RMSF values computed in the 50-150 ns region of the trajectory (C). The protein secondary structure elements are reported as bars (α-helices in blue and β-strands in red). Figure S5. Structure stability of KCTD1BTB closed pentamer throughout the MD simulation: Cα-based RMSD values of trajectory structures computed against the starting model (A), time evolution of the secondary structure content (B), Cα-based RMSF values computed in the 50-150 ns region of the trajectory (C). The protein secondary structure elements are reported as bars (α-helices in blue and β-strands in red). Figure S6. Structure stability of KCTD9BTB closed pentamer throughout the MD simulation: Cα-based RMSD values of trajectory structures computed against the starting model (A), time evolution of the secondary structure content (B), Cα-based RMSF values computed in the 50–150 ns region of the trajectory (C). The protein secondary structure elements are reported as bars (α-helices in blue and β-strands in red). Figure S7. Structure stability of KCTD17BTB closed pentamer throughout the MD simulation: Cα-based RMSD values of trajectory structures computed against the starting model (A), time evolution of the secondary structure content (B), Cα-based RMSF values computed in the 50–150 ns region of the trajectory (C). The protein secondary structure elements are reported as bars (α-helices in blue and β-strands in red). Figure S8. Time evolution of the secondary structure content of KCTD1BTB (A) and KCTD16BTB (B) open pentamers throughout the MD simulations. Figure S9. Cα-based RMSFs of KCTD1BTB and KCTD16BTB open pentamers (A). RMSF values have been computed in the 50–200 ns region of the trajectories by averaging the values detected for each chain. The individual values per chain are reported in panels B (KCTD1BTB) and C (KCTD16BTB). The protein secondary structure elements are reported as bars (α-helices in blue and β-strands in red). Figure S10. Time evolution of the number of H-bonds formed by the central domain of the pentamer with the adjacent ones: interface between chains B–C (A) and C–D (B) throughout the MD simulations of KCTD1BTB (black) and KCTD16BTB (red) open pentamers. Figure S11. Structure stability of the KCTD12-16BTB open pentamer throughout the MD simulation: time evolution of the secondary structure content (A), Cα-based RMSD values of trajectory structures against the starting model (B), Cα-based RMSF values computed in the equilibrated region of the trajectory (50–150 ns) for KCTD16BTB chains (C) and for KCTD12BTB chains (D). The protein secondary structure elements are reported as bars (α-helices in blue and β-strands in red). Figure S12. Structural superimposition of the two crystallographic structures of KCTD16BTB domain in complex with the peptide corresponding to the C-terminal domain of the human GABAB2 receptor. The structure containing the GABAB2R peptide encompassing residues 895-909 is in cyan (PDB ID: 6OCP) [28] whereas the structure containing a longer portion of the peptide (residues 881-913) is in red (PDB ID: 6M8R) [26]. Figure S13. Structure stability of the complex formed by KCTD16BTB and GABAB2R peptide (residues 881-913) throughout the MD simulation: Cα-based RMSD values of trajectory structures against the starting model (B), time evolution of the secondary structure content of GABAB2R peptide (B) and KCTD16BTB pentamer (C), Cα-based RMSF values computed in the 50–150 ns region of the trajectory for the individual KCTD16BTB chains (D). The protein secondary structure elements are reported as bars (α-helices in blue and β-strands in red). Figure S14. Cartoon representation of the crystal structure of the complex between KCTD16BTB and GABAB2 peptide (PDB ID: 6M8R) used as starting model in the MD simulation. Time evolution of the distances between pairs of atoms involved in the formation of H-bonding interactions in the complex. These H-bonds (Val35N-Val910O, Glu28Oε2-Ser913O, Glu102Oε2-Tyr903Oη, Ser69N-Arg891O, Ser69Oγ-Gln889Oε1, Asp91Oδ1-Glu886Oε12, and Asp91Oδ2-Arg890Nη1) that are present in the starting crystallographic model are either sporadic or lost in the simulation. Plots are colored to identify the chains of KCTD16BTB that interact with the peptide (in grey). Figure S15. Cα-based RMSD values of trajectory structures computed against the starting fully extended model of GABAB2R peptide (residues 881-913) (black) and against the crystallographic model extracted from the complex with KCTD16BTB (PDB ID: 6M8R, grey) (A). Cα-based RMSD values computed against the crystallographic GABAB2R peptide structure by considering the helical residues 885-890/885-888 (B) or 899-903/900-903 (C). Figure S16. Autocorrelation function of potential energy (ACF) computed for all MD simulations performed in this study.
