# Supplementary material for: The Structural Versatility of the BTB Domains of KCTD Proteins and Their Recognition of the GABAB Receptor
Source: Biomolecules. 2019 Jul 31;9(8):323. doi: 10.3390/biom9080323 (PMC6722564; doi:10.3390/biom9080323)
Supplement: Supplementary file 1 [file biomolecules-09-00323-s001.pdf]

Supplementary Material

The structural versatility of the BTB domains of KCTD proteins and their recognition of the GABA$_B$ receptor

Nicole Balasco[a,*,#], Giovanni Smaldone[b,#], and Luigi Vitagliano[a,*]

[a]Institute of Biostructures and Bioimaging, CNR, Via Mezzocannone 16, 80134 Napoli, Italy

[b]IRCCS SDN, Via Gianturco 113, 80143 Napoli, Italy

*corresponding authors: Luigi Vitagliano, luigi.vitagliano@unina.it or Nicole Balasco nicole.balasco@unicampania.it

[#] first authors

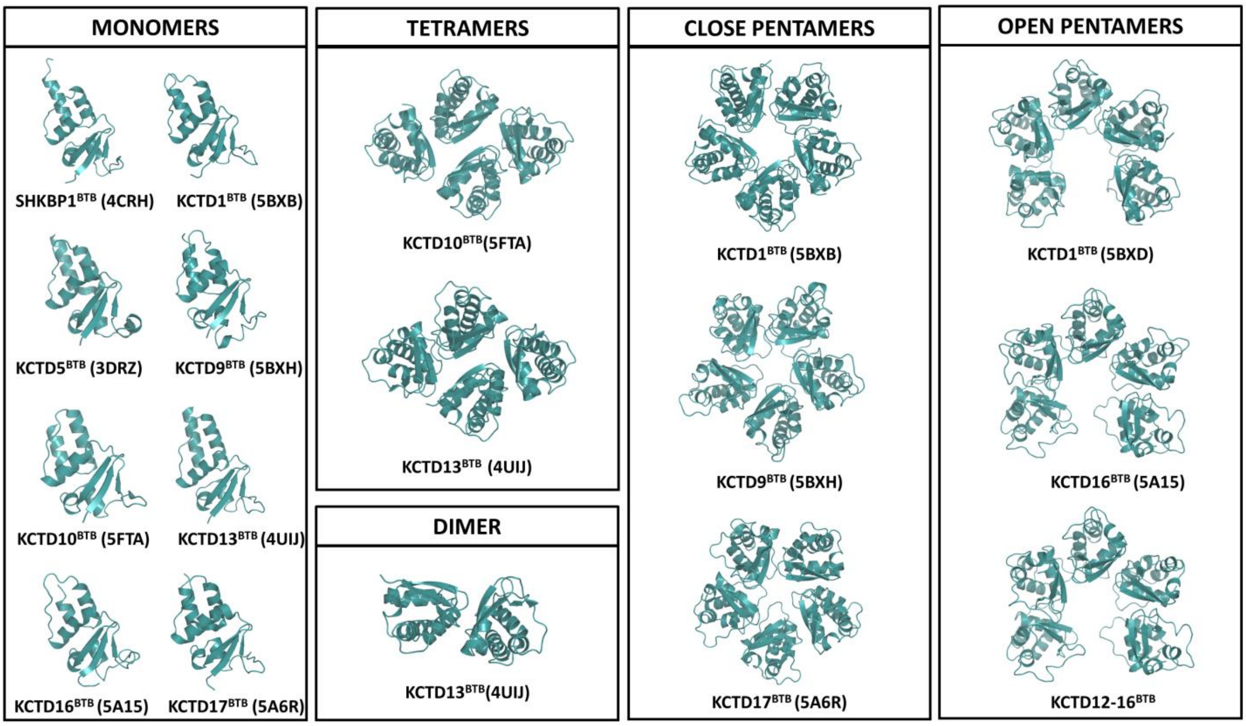

Figure S1. Cartoon representation of the BTB domains of KCTD proteins used as starting models in the MD simulations of monomers, tetramers, dimer, close and open pentamers. PDB codes are reported in bracket.

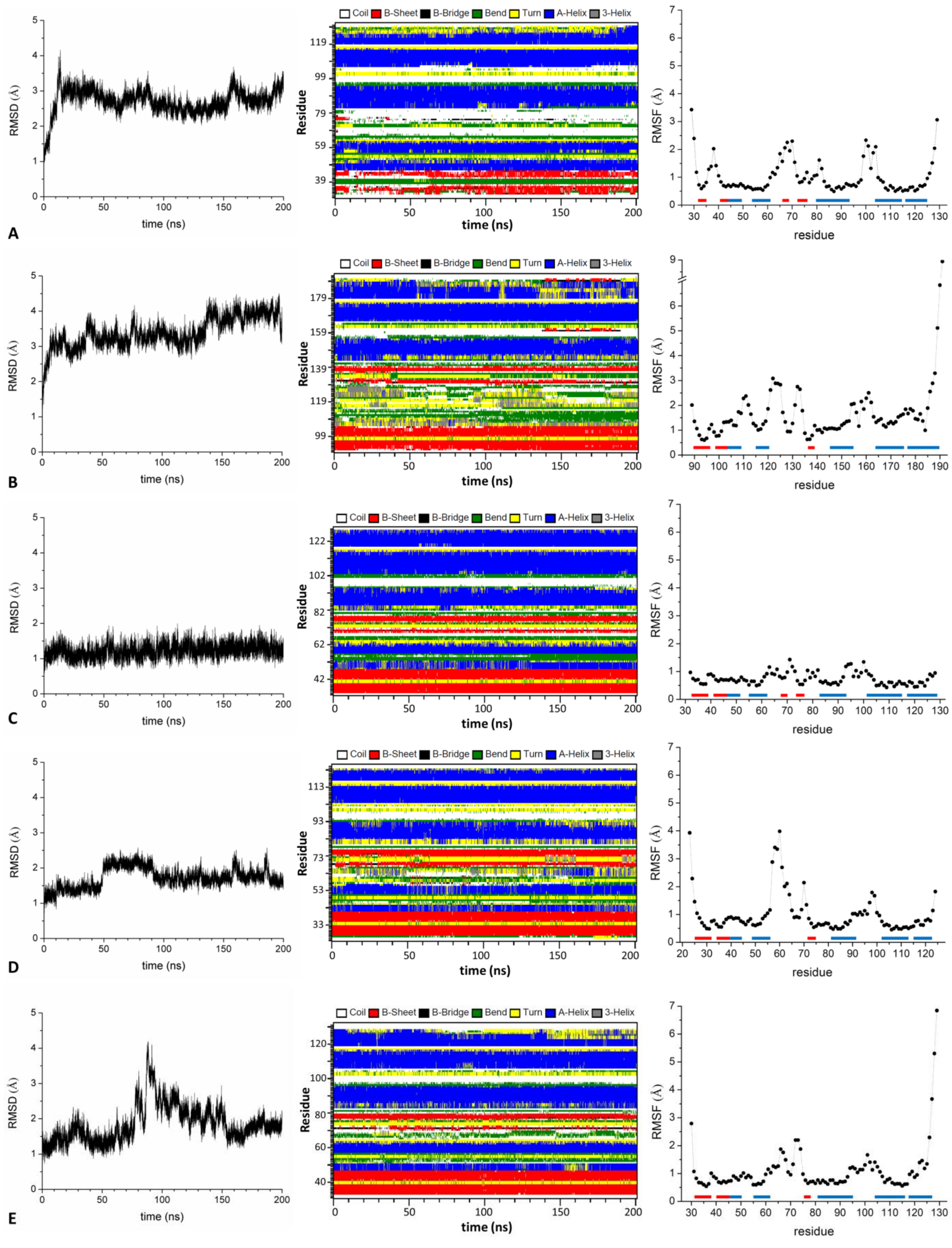

Figure S2. Structure stability of KCTD1^{BTB}(A), KCTD9^{BTB}(B), KCTD10^{BTB}(C), KCTD16^{BTB}(D), and KCTD17^{BTB}(E) monomers throughout the simulations: C$^{\alpha}$-based RMSD values of trajectory structures computed against the starting model, time evolution of the secondary structure content, C$^{\alpha}$-based RMSF values computed in the equilibrated region of the trajectories (50-200 ns). The protein secondary structure elements are reported as bars (α-helices in blue and β-strands in red).

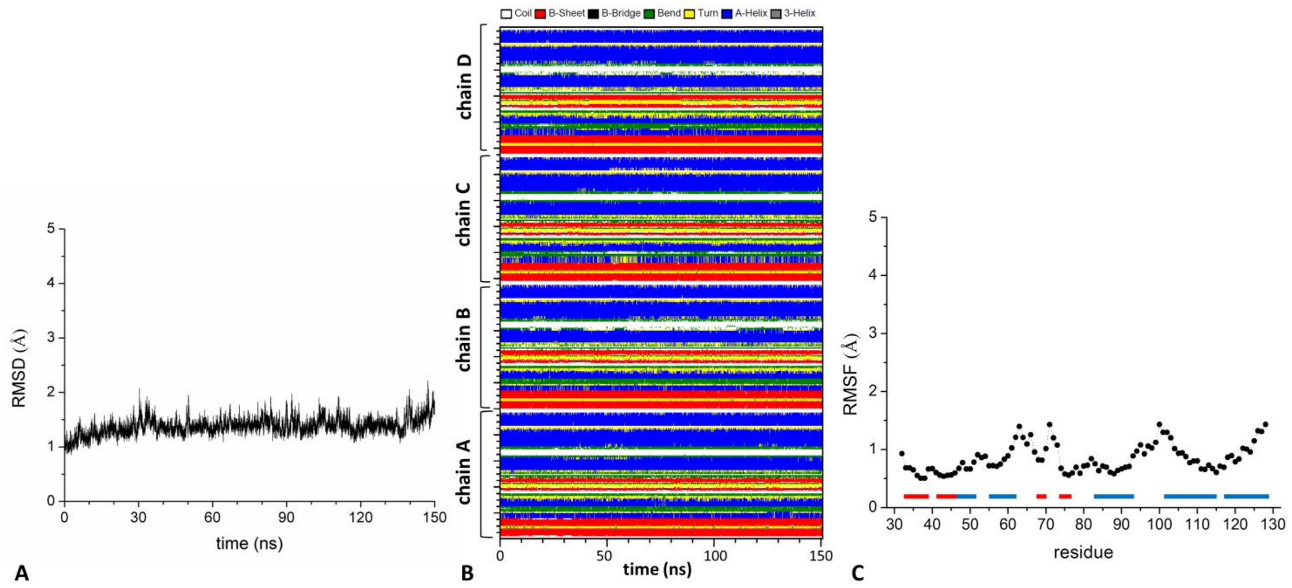

Figure S3. Structure stability of KCTD10^{BTB} tetramer throughout the MD simulation: C$^{\alpha}$-based RMSD values of trajectory structures computed against the starting model (A), time evolution of the secondary structure content (B), C$^{\alpha}$-based RMSF values computed in the 50-150 ns region of the trajectory (C). The protein secondary structure elements are reported as bars (α-helices in blue and β-strands in red).

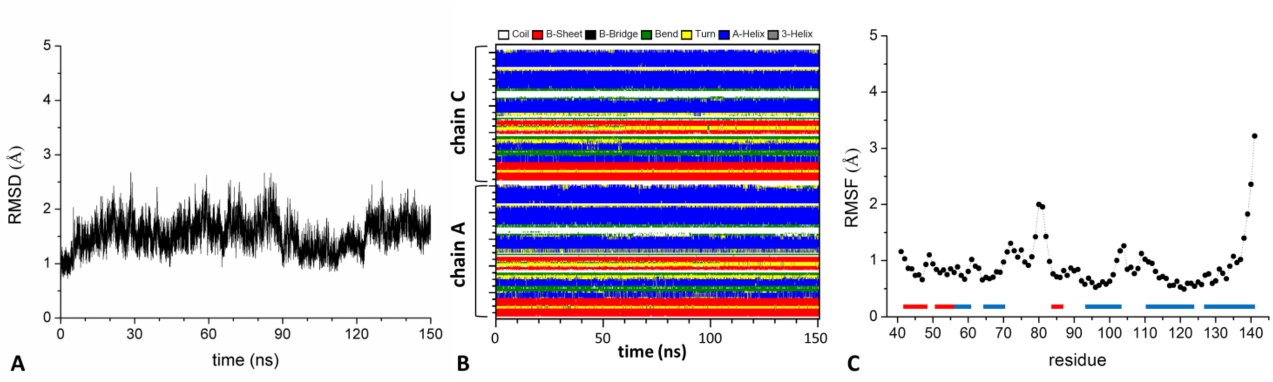

Figure S4. Structure stability throughout the MD simulation of the dimeric building block that constitutes the asymmetric KCTD13^{BTB} tetramer: C$^{\alpha}$-based RMSD values of trajectory structures computed against the starting model (A), time evolution of the secondary structure content (B), C$^{\alpha}$-based RMSF values computed in the 50-150 ns region of the trajectory (C). The protein secondary structure elements are reported as bars (α-helices in blue and β-strands in red).

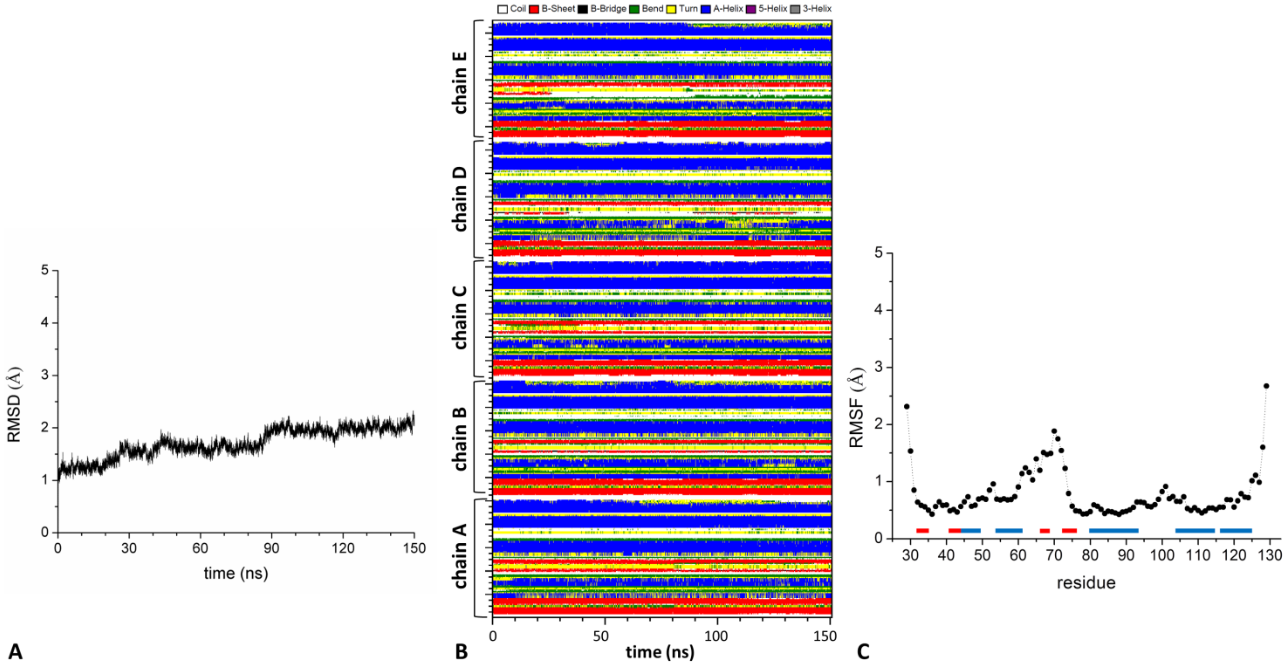

Figure S5. Structure stability of KCTD1^{BTB} closed pentamer throughout the MD simulation: C$^{\alpha}$-based RMSD values of trajectory structures computed against the starting model (A), time evolution of the secondary structure content (B), C$^{\alpha}$-based RMSF values computed in the 50-150 ns region of the trajectory (C). The protein secondary structure elements are reported as bars (α-helices in blue and β-strands in red).

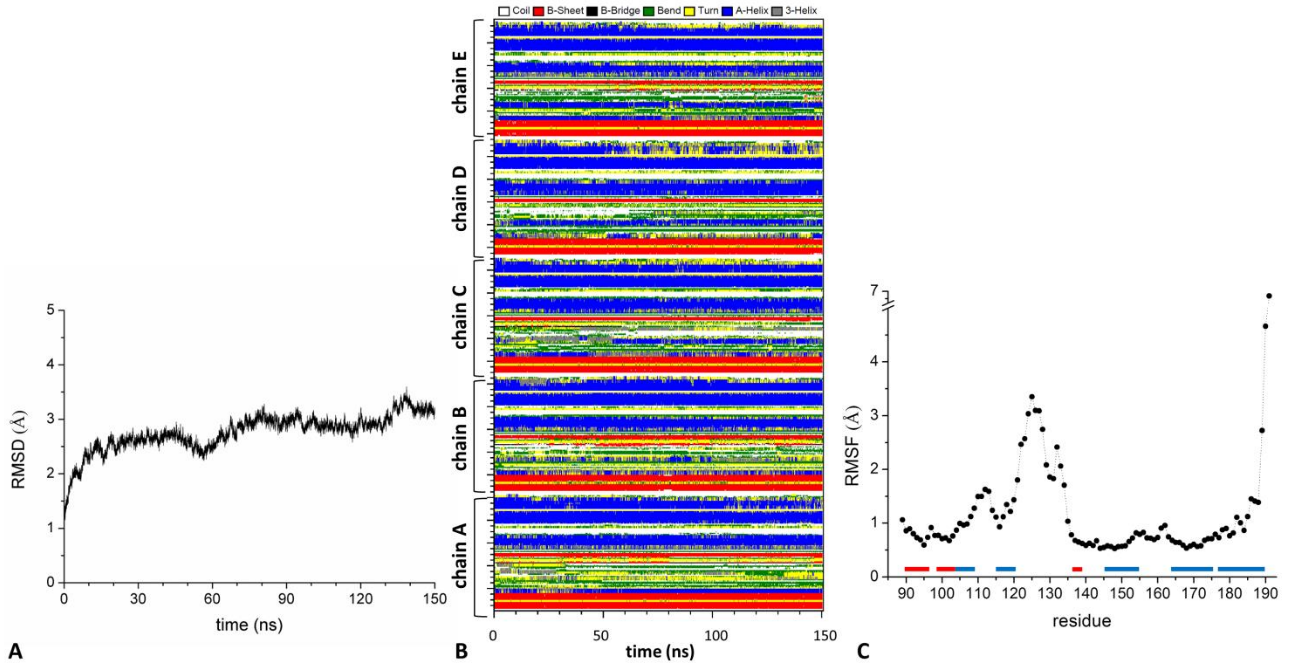

Figure S6. Structure stability of KCTD9^{BTB} closed pentamer throughout the MD simulation: C$^{\alpha}$-based RMSD values of trajectory structures computed against the starting model (A), time evolution of the secondary structure content (B), C$^{\alpha}$-based RMSF values computed in the 50-150 ns region of the trajectory (C). The protein secondary structure elements are reported as bars (α-helices in blue and β-strands in red).

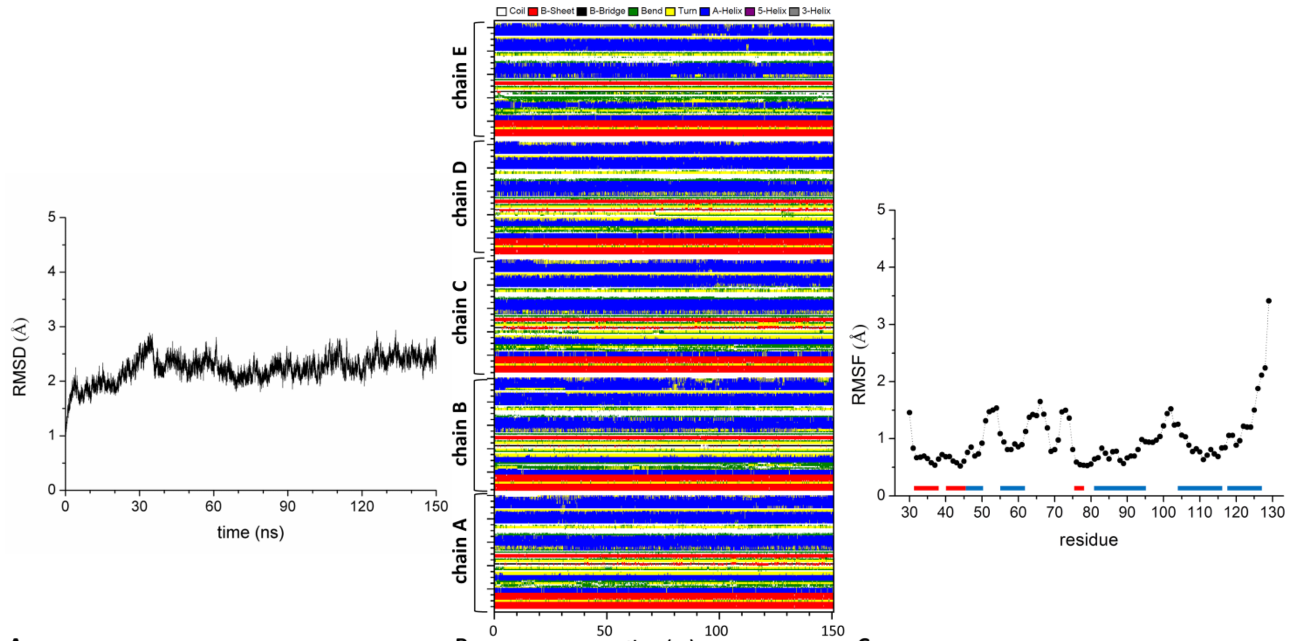

A B C

Figure S7. Structure stability of KCTD17^{BTB} closed pentamer throughout the MD simulation: C$^{\alpha}$-based RMSD values of trajectory structures computed against the starting model (A), time evolution of the secondary structure content (B), C$^{\alpha}$-based RMSF values computed in the 50-150 ns region of the trajectory (C). The protein secondary structure elements are reported as bars (α-helices in blue and β-strands in red).

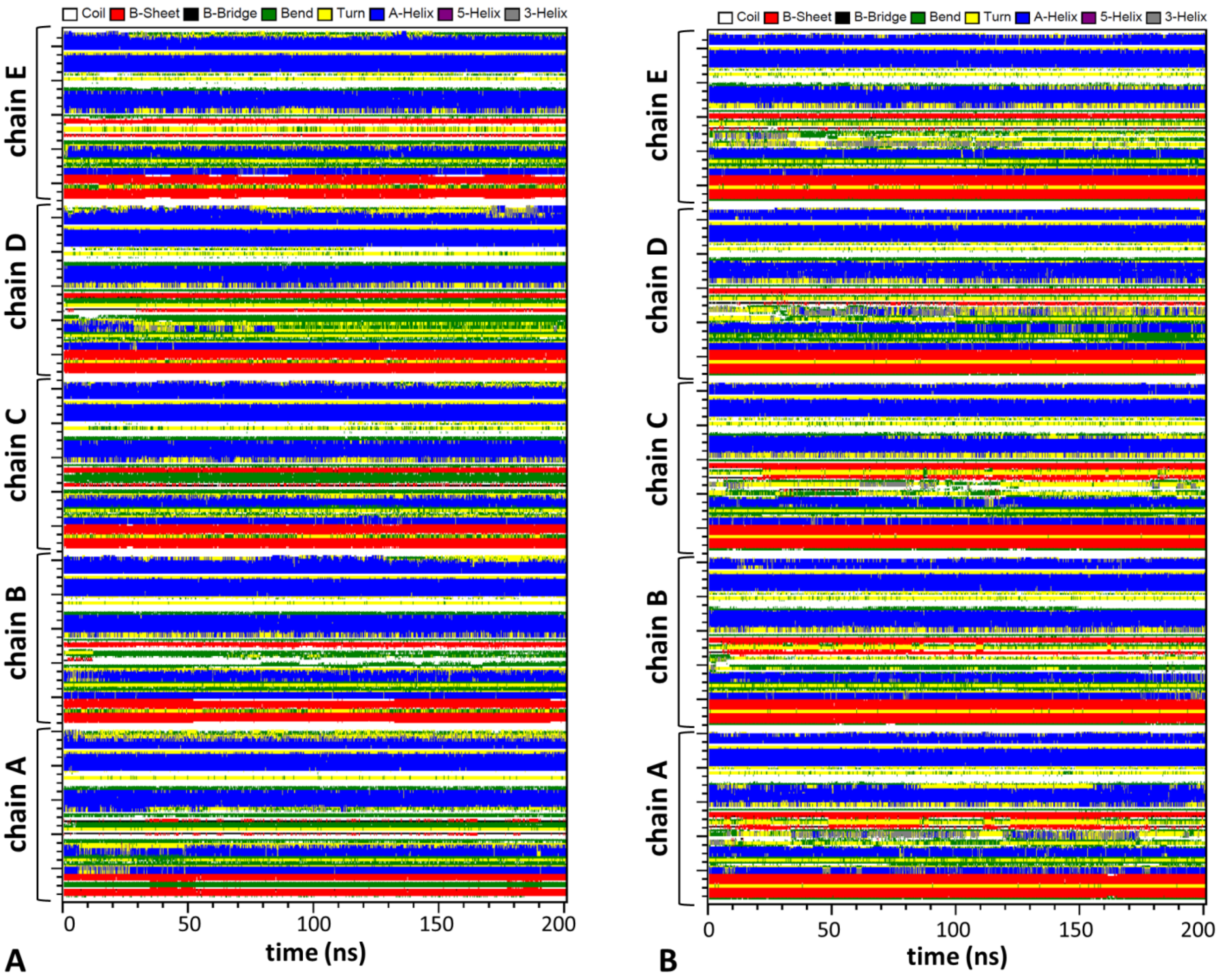

Figure S8. Time evolution of the secondary structure content of KCTD1^{BTB} (A) and KCTD16^{BTB} (B) open pentamers throughout the MD simulations.

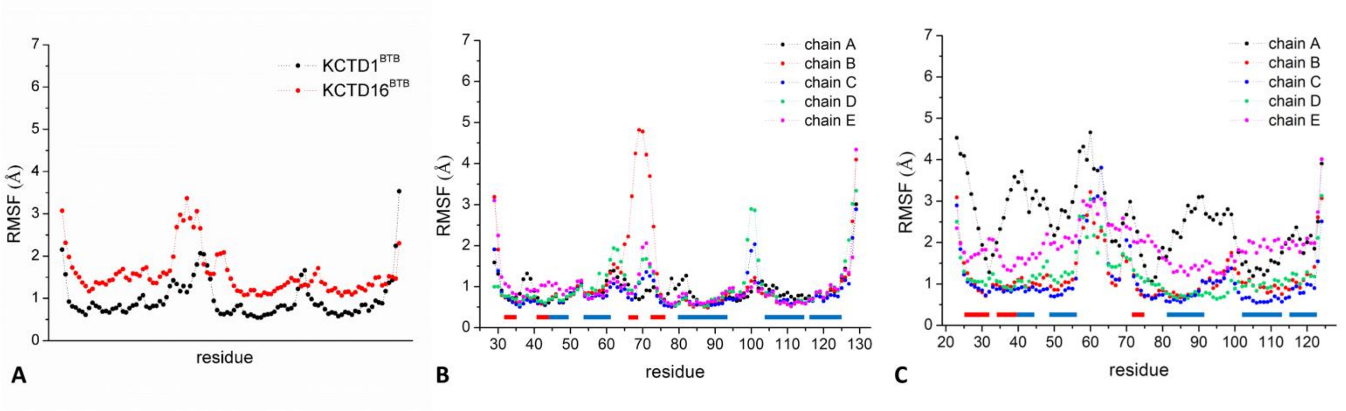

Figure S9. C^{α}-based RMSFs of KCTD1^{BTB} and KCTD16^{BTB} open pentamers (A). RMSF values have been computed in the 50-200 ns region of the trajectories by averaging the values detected for each chain. The individual values *per* chain are reported in panels B (KCTD1^{BTB}) and C (KCTD16^{BTB}). The protein secondary structure elements are reported as bars (α-helices in blue and β-strands in red).

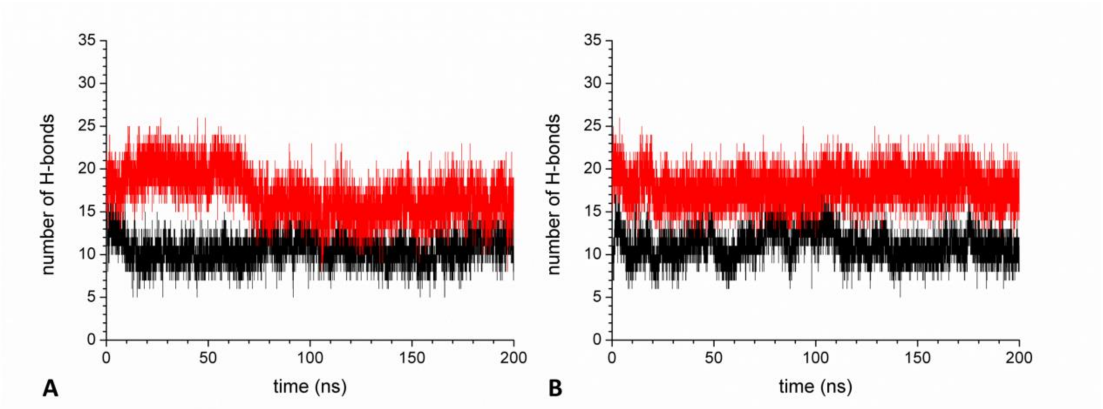

Figure S10. Time evolution of the number of H-bonds formed by the central domain of the pentamer with the adjacent ones: interface between chains B-C (A) and C-D (B) throughout the MD simulations of KCTD1^{BTB} (black) and KCTD16^{BTB} (red) open pentamers.

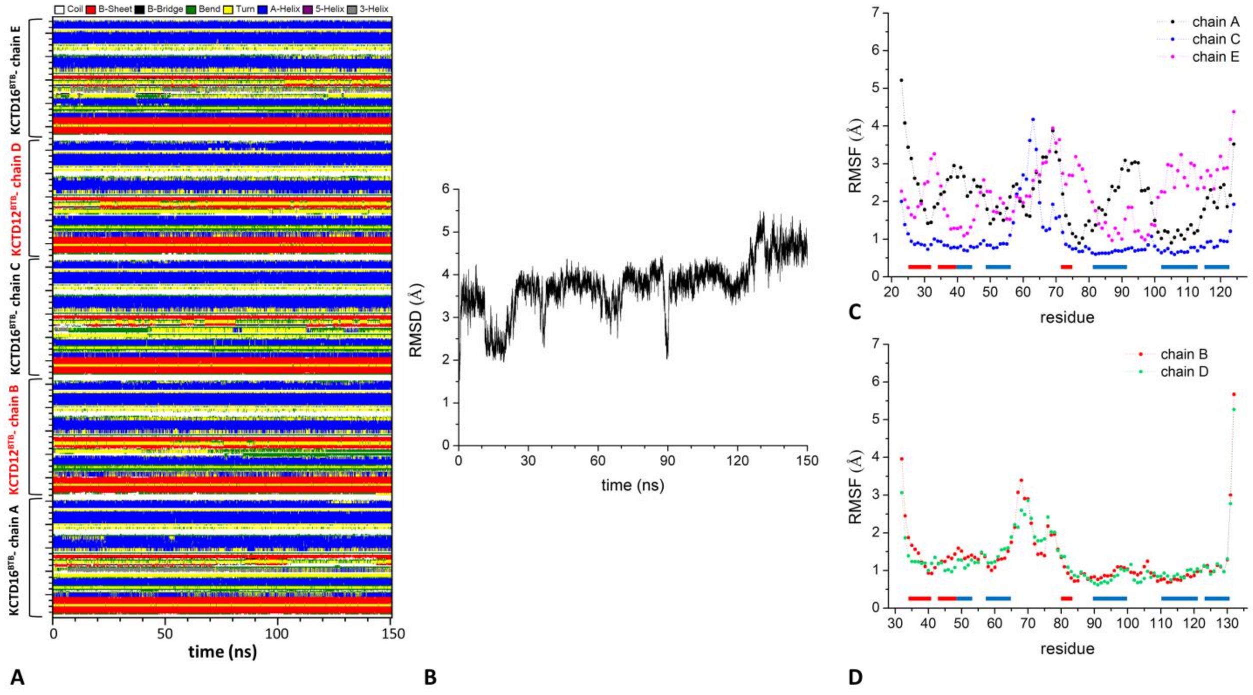

Figure S11. Structure stability of the KCTD12-16^{BTB} open pentamer throughout the MD simulation: time evolution of the secondary structure content (A), C$^{\alpha}$-based RMSD values of trajectory structures against the starting model (B), C$^{\alpha}$-based RMSF values computed in the equilibrated region of the trajectory (50-150 ns) for KCTD16^{BTB} chains (C) and for KCTD12^{BTB} chains (D). The protein secondary structure elements are reported as bars (α-helices in blue and β-strands in red).

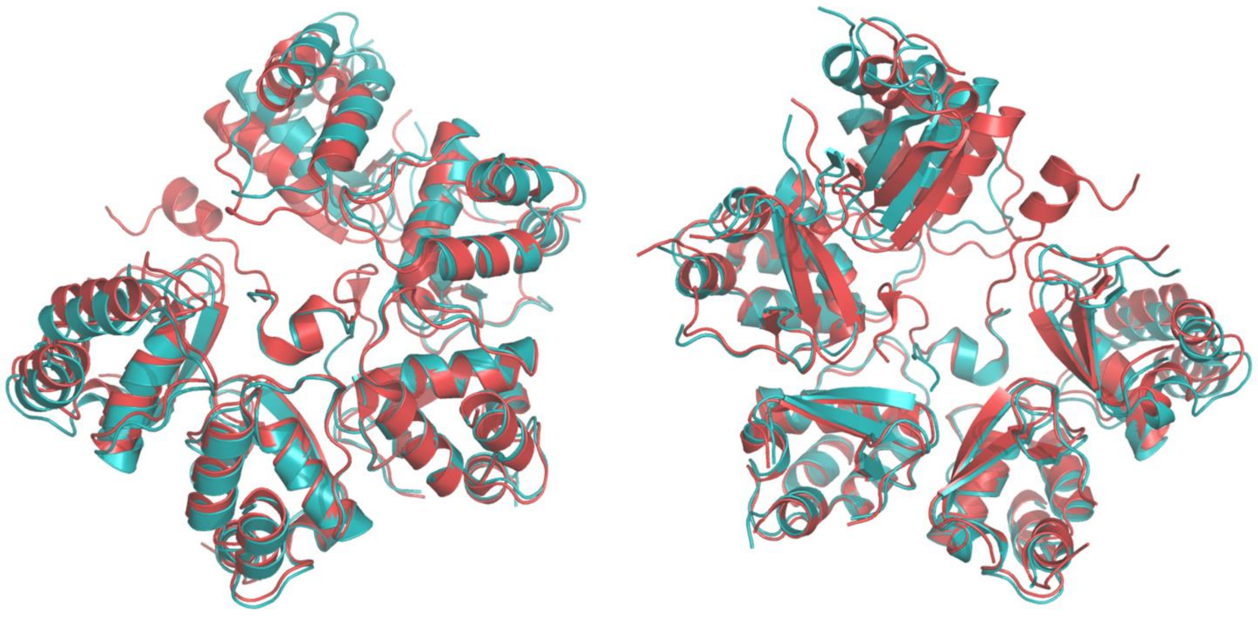

Figure S12. Structural superimposition of the two crystallographic structures of KCTD16^{BTB} domain in complex with the peptide corresponding to the C-terminal domain of the human $GABA_{B2}$ receptor. The structure containing the $GABA_{B2}$R peptide encompassing residues 895-909 is in cyan (PDB ID: 6OCP) [28] whereas the structure containing a longer portion of the peptide (residues 881-913) is in red (PDB ID: 6M8R) [26].

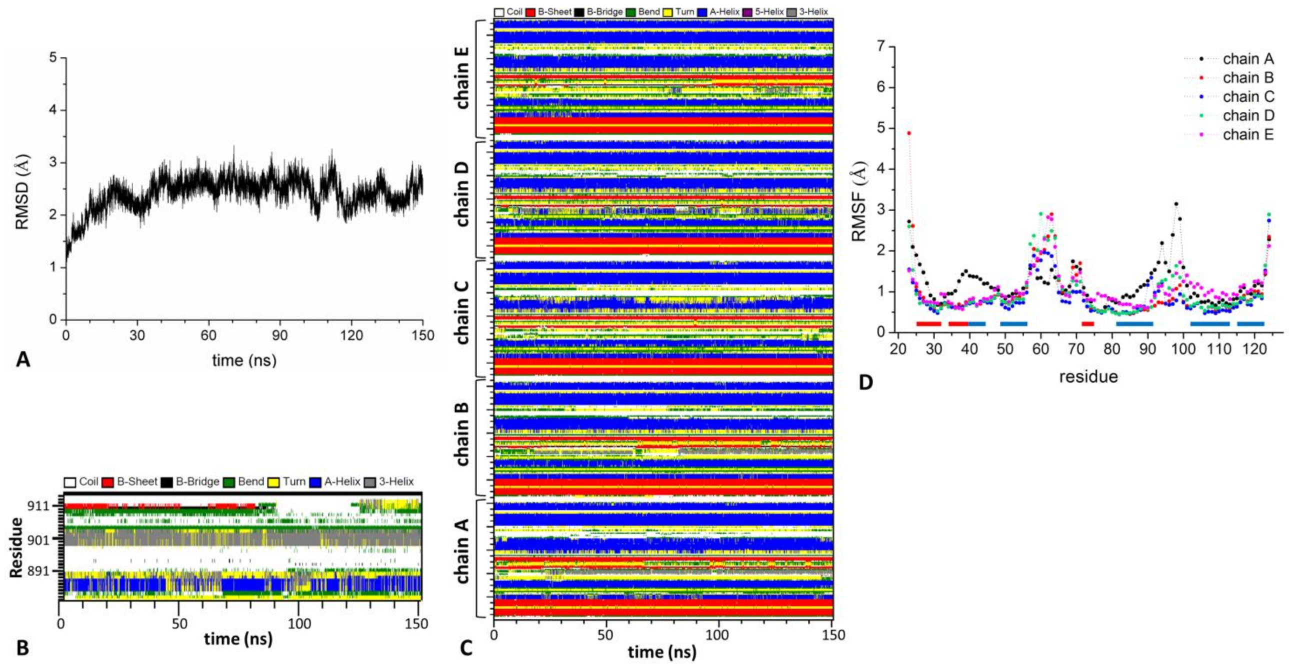

Figure S13. Structure stability of the complex formed by KCTD16^{BTB} and GABA$_{B2}$R peptide (residues 881-913) throughout the MD simulation: C$^{\alpha}$-based RMSD values of trajectory structures against the starting model (B), time evolution of the secondary structure content of GABA$_{B2}$R peptide (B) and KCTD16^{BTB} pentamer (C), C$^{\alpha}$-based RMSF values computed in the 50-150 ns region of the trajectory for the individual KCTD16^{BTB} chains (D). The protein secondary structure elements are reported as bars (α-helices in blue and β-strands in red).

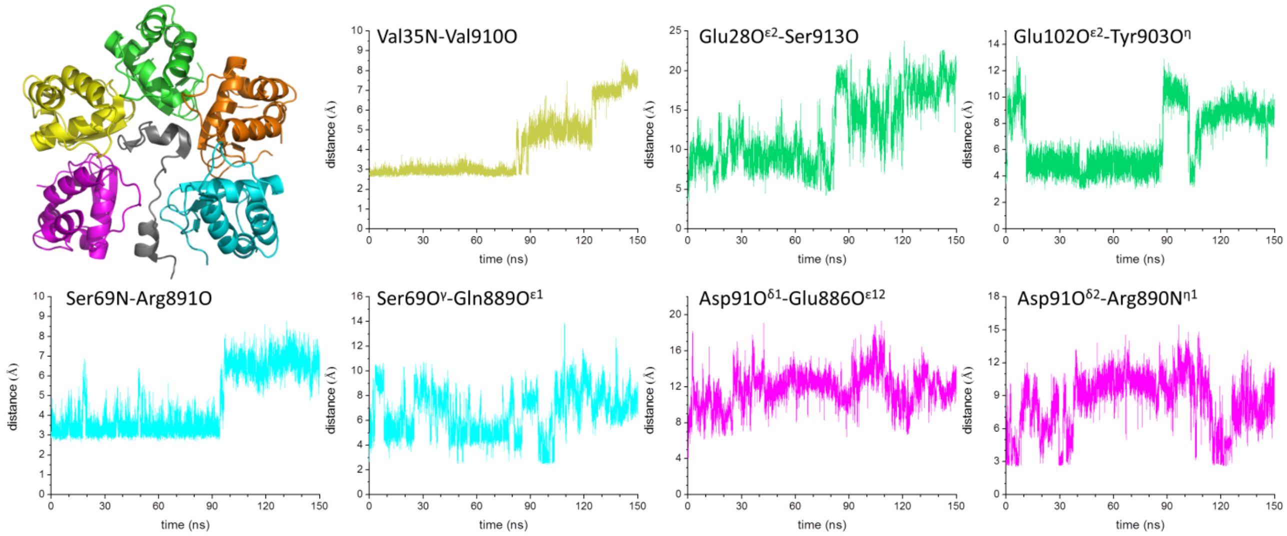

Figure S14. Cartoon representation of the crystal structure of the complex between KCTD16^{BTB} and GABA$_{B2}$ peptide (PDB ID: 6M8R) used as starting model in the MD simulation. Time evolution of the distances between pairs of atoms involved in the formation of H-bonding interactions in the complex. These H-bonds (Val35N-Val910O, Glu28O$^{\varepsilon2}$-Ser913O, Glu102O$^{\varepsilon2}$-Tyr903O$^{\eta}$, Ser69N-Arg891O, Ser69O$^{\gamma}$-Gln889O$^{\varepsilon1}$, Asp91O$^{\delta1}$-Glu886O$^{\varepsilon12}$, and Asp91O$^{\delta2}$-Arg890N$^{\eta1}$) that are present in the starting crystallographic model are either sporadic or lost in the simulation. Plots are colored to identify the chains of KCTD16^{BTB} that interact with the peptide (in grey).

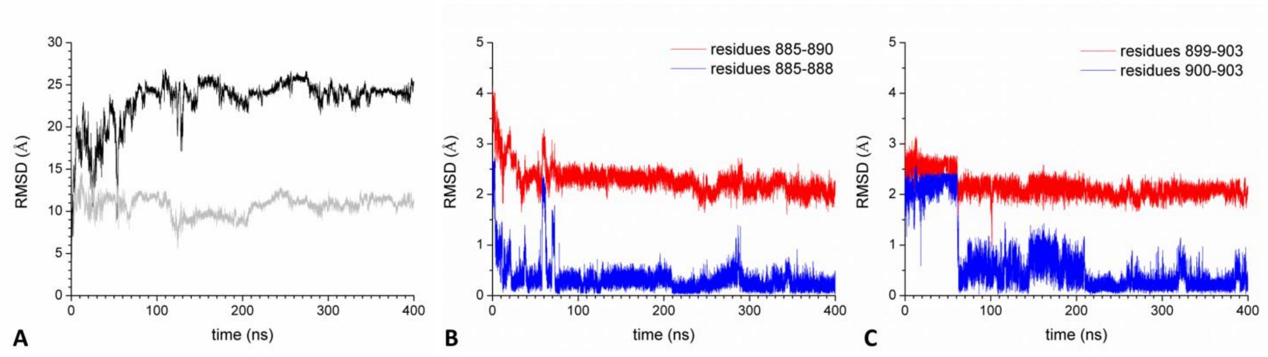

Figure S15. C^{α}-based RMSD values of trajectory structures computed against the starting fully extended model of $GABA_{B2}R$ peptide (residues 881-913) (black) and against the crystallographic model extracted from the complex with $KCTD16^{BTB}$ (PDB ID: 6M8R, grey) (A). C^{α}-based RMSD values computed against the crystallographic $GABA_{B2}R$ peptide structure by considering the helical residues 885-890/885-888 (B) or 899-903/900-903 (C).

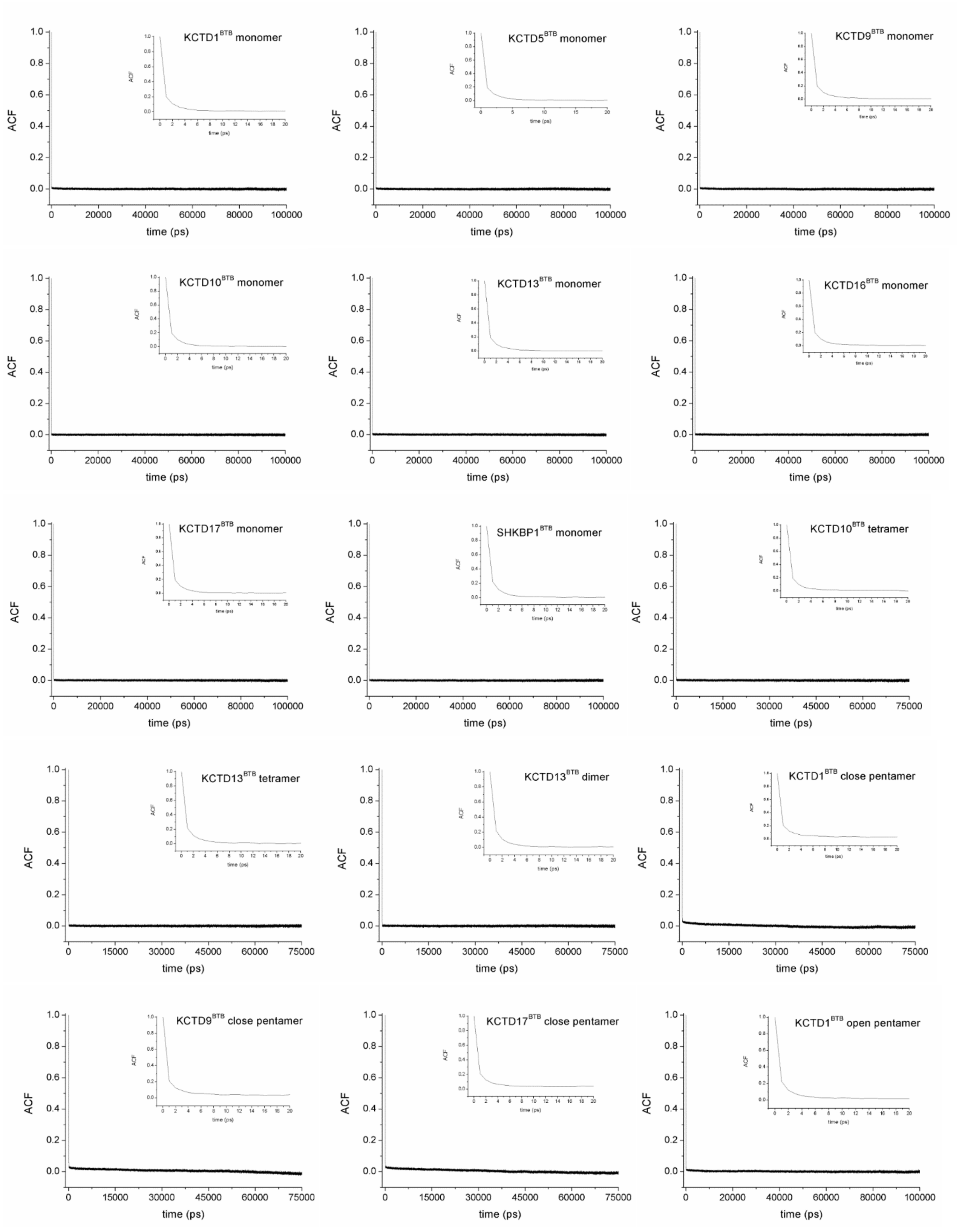
KCTD1^{BTB} monomer
KCTD5^{BTB} monomer
KCTD9^{BTB} monomer
KCTD10^{BTB} monomer
KCTD13^{BTB} monomer
KCTD16^{BTB} monomer
KCTD17^{BTB} monomer
SHKBP1^{BTB} monomer
KCTD10^{BTB} tetramer
KCTD13^{BTB} tetramer
KCTD13^{BTB} dimer
KCTD1^{BTB} close pentamer
KCTD9^{BTB} close pentamer
KCTD17^{BTB} close pentamer
KCTD1^{BTB} open pentamer
ACF
time (ps)

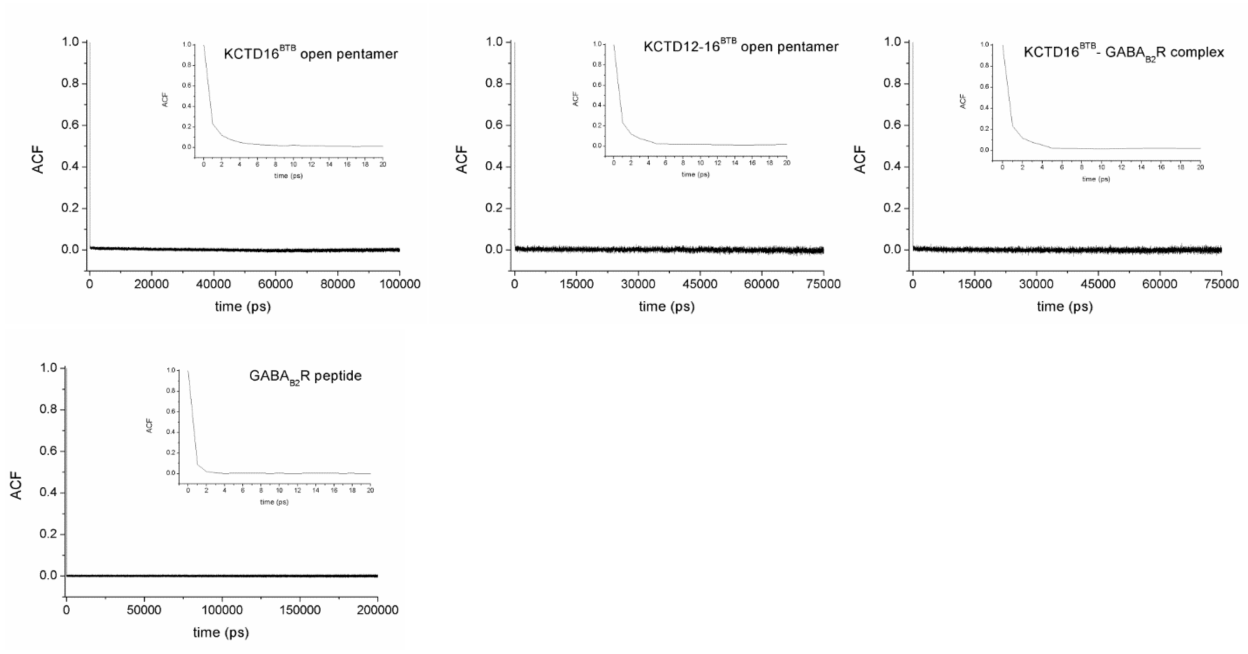

Figure S16. Autocorrelation function of potential energy (ACF) computed for all MD simulations performed in this study.
